# A neutrotrophic trigeminal ulceration

**DOI:** 10.1016/j.jdcr.2025.07.039

**Published:** 2025-10-27

**Authors:** Sarah Fleury, Théo Brochet, Lucile Semeria, Guillaume Chaby

**Affiliations:** a Department of Dermatology, Amiens-Picardy University Hospital, Amiens, France; b Department of Dermatology, AP-HP, Cochin Hospital, Paris, France

**Keywords:** facial ulcer, nasal ulceration, neurotrophic ulceration, trigeminal nerve, trigeminal trophic syndrome, Wallenberg syndrome

## Case report

**Fig 1:**
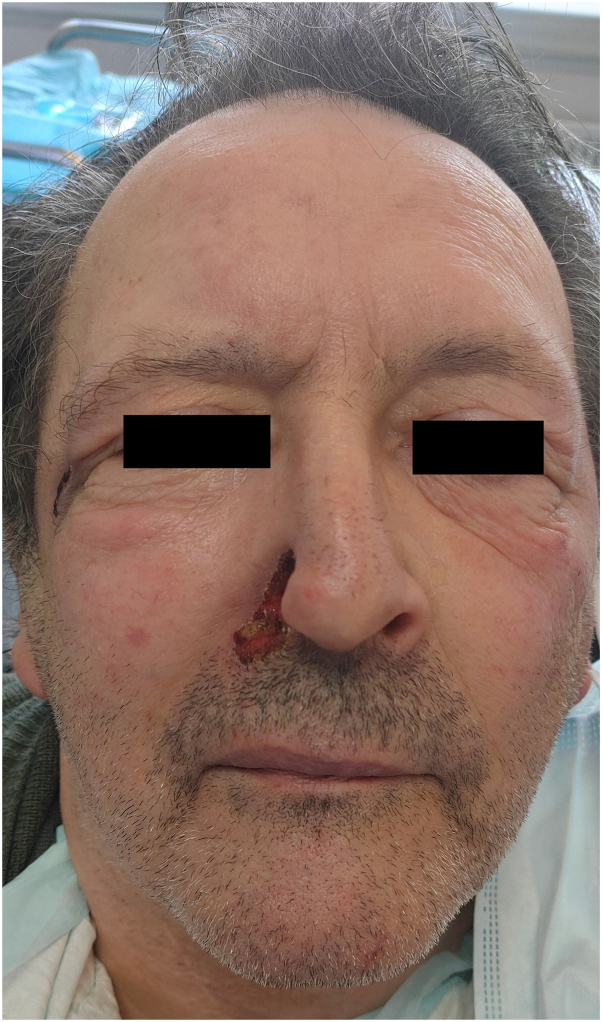
Complete amputation of the right nostril wing.

**Fig 2:**
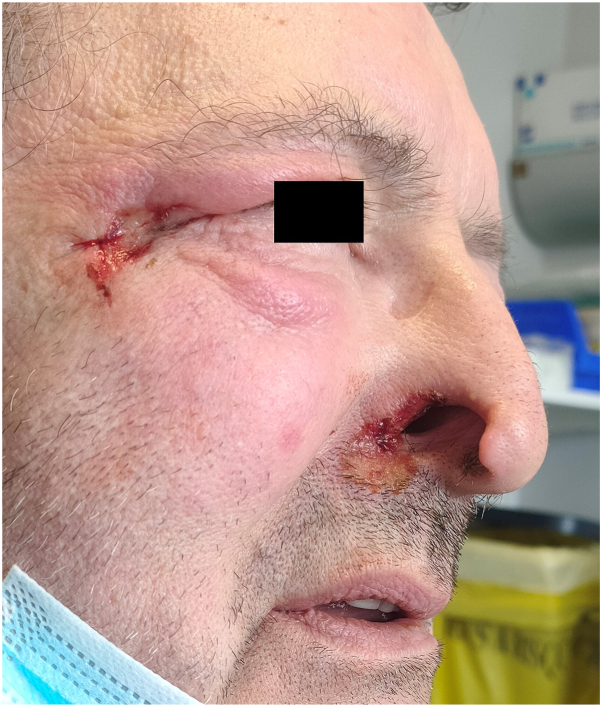
Complete amputation of the right nostril, and ulceration of the right outer canthus.

A 71-year-old man with a history of cerebellar and right bulbar infarction 4 years previously, type 2 diabetes, and hypertension, presented with a deepening ulceration of the right nostril wing, which evolved over 2 years toward complete amputation. A supracentimetric ulceration is also visible near the homolateral external canthus (Figs 1 and 2). A repeated manipulation of this area by the patient is noticed by the family.

A skin biopsy of the right nostril wing, with a local anesthetic painless for the patient, reveals nonspecific inflammatory changes. Skin biopsy of the lesion on the right lateral canthus revealed pre-epitheliomatous keratosis.

The history and clinical presentation led us to diagnose a trigeminal neurotrophic ulceration (TNU) following Wallenberg syndrome, maintained by a scratching tic, with complete anesthesia related to his stroke.

### Question 1: About TNU, what is the correct answer?

A. TNU is associated with hypoesthesia or anesthesia of the trigeminal cutaneous area concerned.
B. It is most often secondary to recurrent herpetic infection.
C. It is usually accompanied by homolateral ocular signs and often associated with factitious skin lesions in other locations.
D. Histologic examination generally reveals an atypical epithelial proliferation mimicking a carcinoma.
E. The therapeutic arsenal may include a short course of systemic corticosteroid therapy.

### Answer:

The correct answer is A.

## Discussion

TNU is a rare cause of facial indolore ulceration, following peripheral or central trigeminal nerve damage. This entity is characterized by skin ulcerations affecting the V1, V2, and V3 dermatomes, which correspond to the three sensory branches of the trigeminal (5th cranial) nerve, following cerebral infarction. It mainly affects women, with an average age of onset of 57 years. The onset of lesions can vary from a few weeks to several decades, mainly on the nostril wing. It is formed by a triad: facial ulceration, anesthesia or hypoesthesia, and dysesthesia in the trigeminal territory.^1^^,^^2^

Neurosurgical procedures, especially Gasser ganglion removal, and strokes involving the posteroinferior cerebellar artery, are the most frequently reported etiologies. Less frequent causes include zona, neurosyphilis, and acoustic neuroma. Skin biopsy is not specific, but essential to rule out differential diagnoses: neoplasia, infections, vasculitis, granulomatosis, pyoderma gangrenosum, and pathomimia.^1^, ^2^, ^3^

The exact mechanism is not precisely known, because not all patients with a trigeminal lesion, whatever the etiology, experience TNU. It appears to be the result of self-induced skin trauma, inconstantly admitted by the patient, caused by continuous rubbing of the area, as a consequence of paresthesia, anesthesia, or dysesthesia.^4^

TNU differs from a factitious disorder, which is generally characterized by multiple localizations and an absence of an underlying sensory disorder. It is not usually acknowledged by the patient. However, it has been suggested that TNU occurs more frequently in patients with underlying psychiatric disorders.^1^ Objective neurologic elements, such as an evocative neurologic context, stereotyped topography, and local anesthesia, help to make the diagnosis.

Management of this pathology is multidisciplinary and not standardized. It includes the prescription of neuroleptics, antidepressants, and antiepileptics. It uses mechanical protection with local devices, such as hydrocolloid dressing. Because of the potential risk of recurrence, psychiatric follow-up is necessary, and cognitive behavioral therapy can help patients to stop manipulations. Local secondary infections may occur.^2^^,^^4^

Other treatment options include transcutaneous electrical neurostimulation, homolateral cervical sympathectomy, stellate ganglionectomy, radiotherapy, and iontophoresis with nerve block. In vitro–cultured epidermal cells could be used in this indication. In very dilapidated forms, as in our patient's case, surgical reconstruction may be proposed.^1^, ^2^, ^3^^,^^5^

Although rare, this condition is important to recognize because of its possible link with neurologic damage, and the serious aesthetic consequences that can result if it is not properly managed.

## Conflicts of interest

None disclosed.
